# Management of skeletal Class III with facial asymmetry using skeletal anchorage: 4-year follow-up

**DOI:** 10.1590/2177-6709.25.2.24.e1-9.onl

**Published:** 2020

**Authors:** Tulika Tripathi, Shilpa Kalra, Priyank Rai

**Affiliations:** 1 Maulana Azad Institute of Dental Sciences, Department of Orthodontics and Dentofacial Orthopaedics (New Delhi, India).

**Keywords:** Maxillary hypoplasia, Facial asymmetry, Skeletal anchorage

## Abstract

**Introduction:**

Skeletal Class III malocclusion with asymmetry is one of the most difficult problems to correct in orthodontics. A functional shift of the mandible in growing patients may occur accompanying a Class III, due to constricted maxillary arch and occlusal interferences. Studies have indicated that posterior unilateral crossbite develops early and has a low rate of spontaneous correction. It may further lead to development of mandibular and facial asymmetry by growth and displacement of mandible if left untreated in growing patients.

**Objective:**

This article reports the clinical case of a thirteen-year-old female patient in CVMI transition stage that had maxillary hypoplasia with a developing facial asymmetry. **Results:** The case was successfully managed with bone-anchored facemask therapy and with elimination of occlusal interferences with guided occlusion. Reverse twin block in the retention phase maintained the results achieved.

**Conclusion:**

A four-year follow-up evaluation revealed successful maintenance of the treatment results.

## RESUMO

**Introdução:**

A má oclusão de Classe III esquelética com assimetria é um dos problemas mais difíceis de se corrigir na Ortodontia. O desvio funcional da mandíbula em pacientes em fase de crescimento pode ocorrer acompanhado da Classe III, devido à atresia maxilar e interferências oclusais. Estudos têm indicado que a mordida cruzada posterior unilateral se desenvolve precocemente e apresenta baixo índice de correção espontânea. Isso pode levar ao desenvolvimento de assimetria mandibular e facial, resultante do crescimento e deslocamento da mandíbula, caso os pacientes não sejam tratados na fase de crescimento.

**Objetivo:**

O presente artigo relata o caso clínico de uma paciente com treze anos de idade, com IMVC em estágio de transição e que apresentava hipoplasia maxilar com assimetria facial em desenvolvimento.

**Resultados:**

O caso foi tratado com sucesso por meio de terapia com máscara facial osseossuportada, eliminando as interferências oclusais por meio de uma oclusão guiada. O uso do aparelho Twin-block reverso como contenção preservou os resultados atingidos.

**Conclusão:**

O acompanhamento após quatro anos confirmou a manutenção dos resultados do tratamento.

## INTRODUCTION

Maxillary hypoplasia in anteroposterior direction in skeletal Class III malocclusion is often accompanied by transverse deficiency of maxilla.^1^ ^,^ ^2^ ^,^ ^3^ Due to the transverse constriction of maxilla, the occlusal interferences exist as the mandible closes into centric occlusion, resulting in functional shift of mandible to one side.^4^ ^,^ ^5^ This leads to the development of a unilateral posterior crossbite. Studies have indicated that posterior unilateral crossbite develops early and has a low rate of spontaneous correction.^6^ ^,^ ^7^ In addition, functional condylar adaptation occurs in concordance with functional mandibular displacement, which may progress into morphologic asymmetry.^8^ ^,^ ^9^ ^,^ ^10^

Treatment of such a case is considered to be challenging, and it requires close observation, with accurate diagnosis and prompt intervention in growing age.

The present article reports the treatment and four-year follow-up of a thirteen-year-old female patient during cervical vertebrae maturity^11^ transition stage, who presented maxillary hypoplasia, with a developing facial asymmetry. She had skeletal Class III malocclusion with maxillary retrusion, mandibular deviation to the right side, and a unilateral posterior crossbite. 

## CASE REPORT

### Diagnosis and etiology

**Figure 1:**
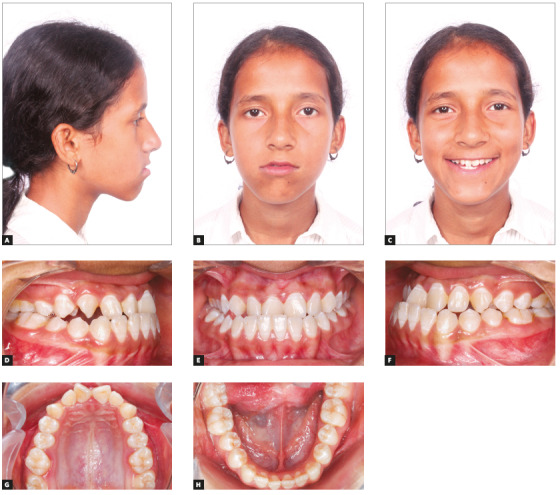
Pretreatment photographs.

A thirteen-year-old female patient presented to the clinical service at the Department of Orthodontics of Maulana Azad Institute of Dental Sciences, with the chief complaint of irregular teeth, and no relevant medical or dental history. On extraoral examination (Figs 1A-C), the patient presented dolichocephalic facial form, with straight profile and straight divergence. Midfacial deficiency with lack of zygomatic prominence was present as well. It was also observed that there was facial asymmetry in the lower third of the face, with deviation of chin to the right side. No symptoms of temporomandibular joint disorder were present. Intraoral examination (Figs 1D-H) revealed a Class III molar relationship on both sides, with posterior crossbite in right premolar/molar region. Mild crowding in both maxillary and mandibular arches was present. Mandibular dental midline was shifted 4.5 mm to the right side, with negative overjet of 1 mm and positive overbite of 3.5 mm. Lateral cephalometric analysis (Table 1 pre-treatment values) revealed a Class III skeletal pattern (ANB = -1°) with a retrognathic maxilla (SNA = 79°, Na perpendicular to Point A = -5mm) and orthognathic but hyperdivergent mandible (SNB = 80°, SND = 78°, Na perpendicular to Pog = -7.5mm, FMA = 39°). The patient had vertical growth pattern (Y axis = 67°) and she was during CVMI transition stage (Stage 3). PA cephalogram confirmed the facial asymmetry in lower third of face, with deviation of Menton point towards right side by 4.5 mm (MSR- Me = +4.5 mm). 

**Table 1:** Cephalometric measurements.

S. No	Cephalometric parameter	Normal (Mean)	Pretreatment	Posttreatment	Postretention
Maxilla					
1.	SNA	82°	79°	82°	82°
2.	Na perp Pt A	0-1 mm	-5 mm	-1 mm	-1.5 mm
Mandible					
3.	SNB	80	80	78	78°
4.	SND	76°	78°	77°	77°
5.	Na perp Pog	-8 to -6 mm	-7.5 mm	-8 mm	-8 mm
Skeletal pattern & Growth pattern					
6.	ANB	2°	-1°	3°	3°
7.	Y-axis	59°	67°	68°	67°
8.	GoGn-SN	32°	36°	38°	39°
9.	FMA	25°	39°	40°	40°
Dentition					
10.	Upper 1 to SN	102°	99°	100°	100°
11.	Upper 1 to NA	22°, 4 mm	18°, 5 mm	22°, 4 mm	21°, 4 mm
12.	Lower 1 to NB	25°, 4 mm	23°, 7 mm	26°, 9 mm	28°, 9 mm
13.	IMPA	90°	84°	86°	88°
Soft tissue					
14.	Upper lip to E line	-4mm	-6 mm	-4 mm	-4.5 mm
15.	Lower lip to E line	-2mm	+1 mm	-1 mm	-2 mm
PA cephalogram					
16.	MSR to Menton (PA Cephalometric value)	0	+4.5 mm to right	+2 mm to right	+2 mm to right

The case was diagnosed as a Class III skeletal malocclusion with retrognathic maxilla and orthognathic mandible. She had vertical growth pattern and facial asymmetry in lower third of face, with chin deviated 4.5 mm to right. As for the occlusal features, the patient had an Angle Class III type 3 malocclusion, with posterior crossbite in right premolar/molar region. The patient had midface deficiency, with lack of zygomatic prominence and retrusive upper lip.

### Treatment objectives

The following treatment goals were established:

1. To correct the skeletal discrepancy and asymmetry.
2. To restrain the vertical growth pattern.
3. To relieve crowding in maxillary arch.
4. To achieve normal overjet and overbite.
5. To achieve stable molar relationship and occlusion on both sides.
6. To improve the soft tissue profile.

### Treatment alternatives

For such a patient in transition stage and vertical growth pattern, conventional facemask therapy is skeptical in terms of its outcome.

Considering the patient’s biological age, the Class III profile with deficient maxilla and facial asymmetry, it was decided for maxillary protraction with bone-anchored protraction facemask therapy, to enhance the use of remaining growth potential at the transition stage. Since most of the mandibular asymmetries in growing Class III cases are due to bilateral constriction of maxilla, it was decided to eliminate the constriction of maxilla by rapid maxillary expansion prior to facemask therapy and hence, stop the developing mandibular asymmetry. Camouflage treatment option was not considered for this patient, because it would not treat the true maxillary hypoplasia and would not result in facial fullness in the middle third of face.^12^ Also, the mandibular asymmetry would not have been addressed. Surgical treatment option was excluded because of the patient’s biological age and her unwillingness for a future major surgical intervention.

### Treatment progress

The treatment duration and the surgical procedure were explained to the patient and her parents, along with the expected outcome, treatment alternatives and retention plan. Then, a written consent was obtained.

**Figure 2:**
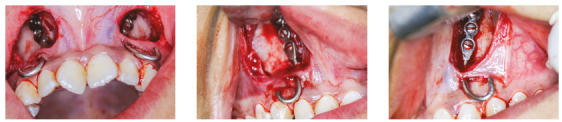
Surgical miniplates in lateral nasal wall area.

Titanium-coated miniplates based on the design used by Kircelli and Pekta^13^ were placed in the lateral nasal wall area by an oral and maxillofacial surgeon (Fig 2). The miniplates were first meticulously contoured to the bilateral nasal walls. The straight extensions were bent into a J-hook shape and were made to project into the oral cavity through an incision in the attached gingiva in lateral incisor/canine region, for the purpose of attaching elastics. 

**Figure 3:**
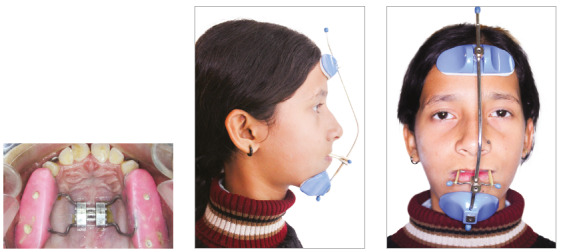
Rapid maxillary expansion and Petit type facemask.

**Figure 4:**
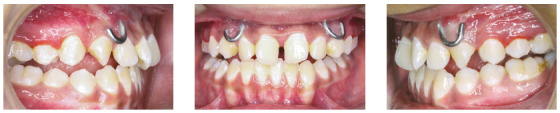
After facemask therapy.

After two weeks of soft tissue healing, rapid maxillary expansion was started. Maxillary protraction with facemask started with force of 8 ounces/side and high-pull chincup wear was advised for a duration of 16 hours/day (Fig 3). Force levels gradually increased to 14 ounces/side. Maxillary protraction was continued for 4 months until achievement of 4mm of positive overjet (Fig 4).

**Figure 5:**
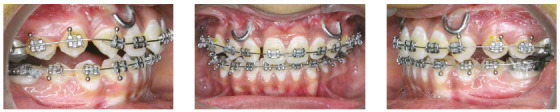
Fixed mechanotherapy.

Facemask therapy was then continued for night-time wear whilst full-fixed mechanotherapy was started. Maxillary and mandibular arches were aligned with series of light archwires (Fig 5). Spaces in maxillary arch were consolidated. Class II elastic on right side and Class III elastics (5/16-in, 3.5 ounces) were used for correction of residual midline shift. Triangular settling elastics (1/8-in, 2 ounces) were used for final settling of occlusion. 

**Figure 6:**
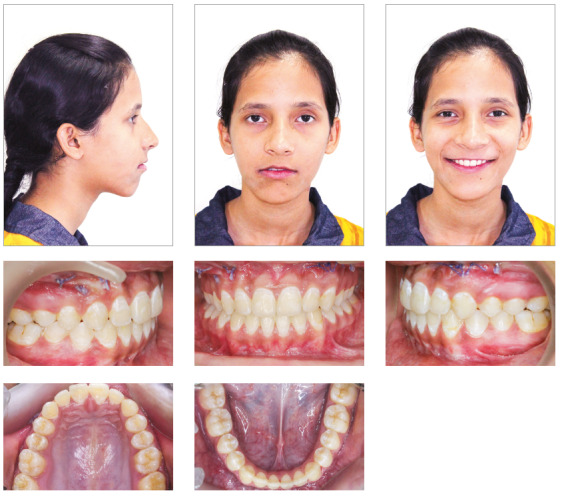
Posttreatment photographs.

**Figure 7:**
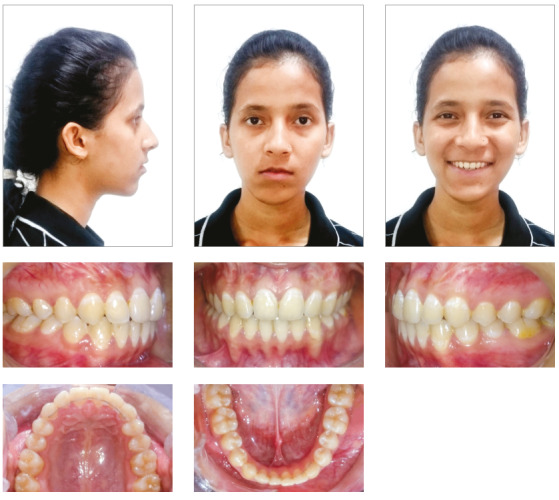
Postretention photographs after 4 years.

**Figure 8:**
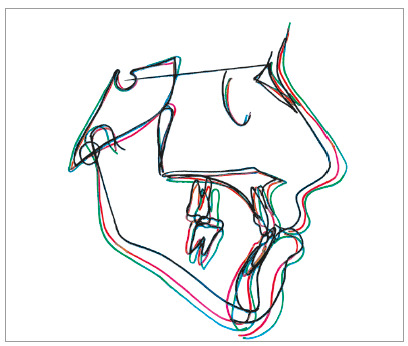
Superimposition SN at S.

Cephalometric analysis revealed significant changes (Table 1, post-treatment values) with significant improvement in soft tissue profile of the patient. The case was debonded after one year of full-fixed mechanotherapy (Fig 6). In retention phase, reverse twin block was prescribed along with high-pull chincup. Four years after retention, the treatment results were maintained, with good soft tissue profile of the patient (Fig 7 and Table 1). Although mandibular dental midline did not coincide with the maxillary midline, the results showed significant improvement in Class III relation and mandibular asymmetry, with only minimal residual dental midline shift, which was clinically acceptable. Stable molar relation with stable occlusion was achieved after treatment, and was maintained even after four years of retention. Mandibular asymmetry improved to a great extent and was within clinically acceptable range. Superimposition also showed significant improvement in skeletal malocclusion and soft tissue profile after treatment, and maintenance of treatment results after four years of retention (Fig 8).

## DISCUSSION

Facemask therapy results in skeletal and dentoalveolar changes. Skeletal changes include maxillary protrusive movement and downward and backward rotation of the mandible, with a decrease in mandibular prognathism. Such changes induce favorable changes in the facial profile. Dentoalveolar changes mainly consist of linguoversion of mandibular incisors and labial inclination of maxillary incisors.^14^ In conventional facemask therapy, the forces used for maxillary protraction are usually applied to maxillary teeth. Hence, undesirable side-effects such as anterior rotation of the maxilla, proclination of the maxillary incisors, excessive dental forward movement, and extrusion of the maxillary molars due to indirect application of force are associated with conventional facemask therapy.^15^ ^-^ ^17^ These effects might camouflage the malocclusion and conflict with the main goals of the skeletal Class III treatment. To overcome these undesirable side-effects and achieve true maxillary protraction with direct force application to circum-maxillary sutures, it is desirable to use a protocol with rigid skeletal anchorage. 

Additionally, one of the most important factors for successful maxillary protraction treatment is to determine the optimal timing to start treatment. Most studies suggest that protraction headgear therapy is more effective if implemented during the deciduous and early mixed dentitions.^18,19^ However, maxillary protraction with bone anchors has been reported to be successful in the late mixed or permanent dentition phases.^20^ Since the patient was in CVMI transition stage, and in order to utilize the remaining growth potential at the most, with maximum skeletal effects, skeletally anchored facemask therapy was preferred.

The amount of maxillary protraction in Class III cases that are treated with skeletal anchorage might vary from 3.0 to 5.6mm.^13^ ^,^ ^21^ ^-^ ^23^ It can cause significantly larger maxillary advancement (2.3-3mm more), compared to the conventional facemask therapy.^20^ It also results in fewer vertical changes. Furthermore, these patients do not exhibit clockwise rotation of the mandible or dental compensation.^20^ ^,^ ^24^ The upper lip and lip sulcus also move forward, and the soft tissue B point and pogonion move backward during the protraction period, indicating improvements in the soft tissue profile in line with the underlying skeletal components during the protraction procedure.^25^ ^-^ ^27^

Rapid maxillary expansion with a bonded appliance was performed. It is suggested that expansion appliance enhances the protraction effects in terms of time, with less dental and more skeletal effects. RME can disarticulate circum-maxillary sutures to facilitate the forward movement of the maxilla via facemask therapy and lead to downward and forward movement of A-point.^28^ ^,^ ^29^ RME also eliminated the causative factor for functional shift of mandible, i.e., constriction of maxilla, and resulted in improvement in mandibular asymmetry after expansion and protraction.

In the present case, miniplates were placed in the lateral nasal wall of the maxilla and orthopedic forces were applied directly to the intraoral extensions of the miniplate. The lateral nasal wall area of the maxilla has an advantage of being anterior to the center of resistance of the nasomaxillary complex (the posterosuperior ridge of the pterygomaxillary fissure)^30^, which allows resulting force vector close to the center of resistance and in line with the downward and forward growth of the maxilla.^31^ Furthermore, the lateral nasal wall of the maxilla is the most appropriate anatomic site for achieving the fullness of the nasobuccal folds, the infraorbital region, and consequently, the soft-tissue profile.^13^ ^,^ ^32^ In an animal model, Smalley et al.^4^ used osseointegrated implants to protract the maxillofacial complex, where greatest remodeling took place in the sutures and the bones closest to the force application point. Similarly, the present patient showed remarkable midfacial protraction and had positive improvement of her soft-tissue profile. 

Asymmetry is one of the most difficult problems to correct in orthodontics. Synergistic effect of maxillary protraction and rapid maxillary expansion eliminated the occlusal interferences and resulted in correction of functional shift of mandible, improving the facial asymmetry. Remaining dental asymmetry was corrected by use of diagonal elastics.^6^

## CONCLUSION

The use of skeletally anchored facemask therapy along with rapid maxillary expansion successfully managed the case of skeletal Class III with developing facial asymmetry in CVMI transition stage, which if not treated at this stage would have required a future more complex therapeutic approach, involving dental extractions and/or orthognathic surgery.

## References

[1] Franchi L, Baccetti T (2005). Transverse maxillary deficiency in Class II and Class III malocclusions a cephalometric and morphometric study on posteroanterior films. Orthod Craniofac Res.

[2] Proffit WR, Phillips C, Prewitt JW, Turvey TA (1991). Stability after surgical-orthodontic correction of skeletal Class III malocclusion 2. Maxillary advancement. Int J Adult Orthodon Orthognath Surg.

[3] McNamara JA (2000). Maxillary transverse deficiency. Am J Orthod Dentofacial Orthop.

[4] Smalley WM, Shapiro PA, Hohl TH, Kokich VG, Brånemark PI (1988). Osseointegrated titanium implants for maxillofacial protraction in monkeys. Am J Orthod Dentofacial Orthop.

[5] Joondeph DR (2000). Mysteries of asymmetries. Am J Orthod Dentofacial Orthop.

[6] Bishara SE, Burkey PS, Kharouf JG (1994). Dental and facial asymmetry a review. A Angle Orthod.

[7] Kurol J, Berglund L (1992). Longitudinal study and cost-benefit analysis of the effect of early treatment of posterior crossbites in the primary dentition. Eur J Orthod.

[8] Thilander B, Wahlund S, Lennartsson B (1984). The effect of early interceptive treatment in children with posterior crossbite. Eur J Orthod.

[9] Lindner A, Hendrickson C, Odenrick L, Modeer T (1986). Maxillary expansion of unilateral cross-bite in preschool children. Scand J Dent Res.

[10] Maurice TJ, Kula K (1998). Dental arch asymmetry in the mixed dentition. Angle Orthod.

[11] Baccetti T, Franchi L, McNamara JA (2002). An improved version of the cervical vertebral maturation (CVM) method for the assessment of mandibular growth. Angle Orthod.

[12] Tekale PD, Vakil KK, Vakil JK, Parhad SM (2014). Orthodontic camouflage in skeletal Class III malocclusion A contemporary review. J Orofac Res.

[13] Kircelli BH, Pektas ZO (2008). Midfacial protraction with skeletally anchored face mask therapy A novel approach and preliminary results. Am J Orthod Dentofacial Orthop.

[14] da Silva Filho OG.Magro AC.CapelozzaFilho L (1998). Early treatment of the class III malocclusion with rapid maxillary expansion and maxillary protraction. Am J Orthod Dentofacial Orthop.

[15] Baccetti T, McGill JS, Franchi L, McNamara JA, Tollaro I (1998). Skeletal effects of early treatment of class III malocclusion with maxillary expansion and face mask therapy. Am J Orthod Dentofacial Orthop.

[16] Baik HS (1995). Clinical results of the maxillary protraction in Korean children. Am J Orthod Dentofacial Orthop.

[17] MacDonald KE, Kapust AJ, Turley PK (1999). Cephalometric changes after the correction of class III malocclusion with maxillary expansion/facemask therapy. Am J Orthod Dentofacial Orthop.

[18] Merwin D, Ngan P, Hagg U, Yiu C, Wei SH (1997). Timing for effective application of anteriorly directed orthopedic force to the maxilla. Am J Orthod Dentofacial Orthop.

[19] Westwood PV, McNamara JA, Baccetti T, Franchi L, Sarver DM (2003). Long-term effects of Class III treatment with rapid maxillary expansion and facemask therapy followed by fixed appliance. Am J Orthod Dentofacial Orthop.

[20] Cevidanes L, Baccetti T, Franchi L, McNamara JA, De Clerck H (2010). Comparison of two protocols for maxillary protraction: bone anchors versus face mask with rapid maxillary expansion. Angle Orthod.

[21] Kaya D, Kocadereli I, Kan B, Tasar F (2011). Effects of facemask treatment anchored with miniplates after alternate rapid maxillary expansions and constrictions; a pilot study. Angle Orthod.

[22] De Clerck HJ, Cornelis MA, Cevidanes LH, Heymann GC, Tulloch CJ (2009). Orthopedic traction of the maxilla with miniplates a new perspective for treatment of midface deficiency. J Oral Maxillofac Surg.

[23] Baek SH, Kim KW, Choi JY (2010). New treatment modality for maxillary hypoplasia in cleft patients: protraction facemask with miniplate anchorage. Angle Orthod.

[24] Morales-Fernández M, Iglesias-Linares A, Yañez-Vico RM, Mendoza-Mendoza A, Solano-Reina E (2013). Bone- and dentoalveolar-anchored dentofacial orthopedics for Class III malocclusion new approaches, similar objectives? A systematic review. Angle Orthod.

[25] Yilmaz HN, Garip H, Satilmis T, Kucukkeles N (2015). Corticotomy-assisted maxillary protraction with skeletal anchorage and Class III elastics. Angle Orthod.

[26] De Clerck H, Cevidanes L, Baccetti T (2010). Dentofacial effects of bone-anchored maxillary protraction a controlled study of consecutively treated class III patients. Am J Orthod Dentofacial Orthop.

[27] Sar C, Arman-Özçirpici A, Uçkan S, Yazici AC (2011). Comparative evaluation of maxillary protraction with or without skeletal anchorage. Am J Orthod Dentofacial Orthop.

[28] Tanne K, Hiraga J, Sakuda M (1989). Effects of directions of maxillary protraction forces on biomechanical changes in craniofacial complex. Eur J Orthod.

[29] Yu HS, Baik HS, Sung SJ, Kim KD, Cho YS (2007). Three-dimensional finite-element analysis of maxillary protraction with and without rapid palatal expansion. Eur J Orthod.

[30] Kambara T (1977). Dentofacial changes produced by extraoral forward force in the Macacairus. Am J Orthod.

[31] McNamara JA (1987). An orthopedic approach to the treatment of Class III malocclusion in young patients. J Clin Orthod.

[32] Tripathi T, Rai P, Singh N, Kalra S (2016). A comparative evaluation of skeletal, dental, and soft tissue changes with skeletal anchored and conventional facemask protraction therapy. J Orthod Sci.

